# Short sleep duration and incident self-reported severe periodontitis among Japanese adults: a nationwide prospective cohort study

**DOI:** 10.3389/fpubh.2026.1946230

**Published:** 2026-09-30

**Authors:** Noriko Takeuchi, Kanon Itoi, Takayuki Maruyama, Takahiro Tabuchi, Daisuke Ekuni

**Affiliations:** 1 Department of Preventive Dentistry, Division of Dentistry, Medical Development Field, Okayama University, Okayama, Japan; 2 Dental School, Okayama University, Okayama, Japan; 3 Department of Preventive Dentistry, Faculty of Medicine, Dentistry and Pharmaceutical Sciences, Okayama University, Okayama, Japan; 4 Division of Epidemiology, School of Public Health, Tohoku University Graduate School of Medicine, Sendai, Japan

**Keywords:** cohort study, oral health, periodontitis, prospective study, public health, sleep duration

## Abstract

**Background:**

Periodontitis is a chronic inflammatory condition influenced by behavioral, systemic and socioeconomic factors, and its prevalence increases with age. Although cross-sectional studies suggest an association between short sleep duration and periodontitis, longitudinal evidence remains limited. This study aimed to examine the association between sleep duration and self-reported incident severe periodontitis using data from a nationwide prospective cohort.

**Methods:**

We conducted a prospective cohort study using longitudinal data from the 2023–2025 waves of the Japan COVID-19 and Society Internet Survey. Participants without self-reported severe periodontitis at baseline were followed for self-reported incident severe periodontitis. Short sleep duration was defined as <6 h per night. Cox proportional hazards models were used as the primary analysis, and generalized estimating equations were used as a secondary analysis.

**Results:**

Among 33,000 respondents to the 2023 survey, 8,144 individuals without self-reported severe periodontitis at baseline and with follow-up data were included in the study population. Short sleep duration was reported by 22.7% of participants and was associated with a higher risk of self-reported incident severe periodontitis (hazard ratio = 1.28, 95% confidence interval [CI]: 1.13–1.46). This association was consistent in the secondary analysis using repeated measures (odds ratio = 1.26, 95% CI: 1.09–1.46). Older age, lower income, diabetes, dyslipidaemia, depression and smoking were also associated with an increased risk.

**Conclusion:**

Short sleep duration may be a modifiable risk factor for self-reported incident severe periodontitis in the general population. The present findings highlight the potential importance of sleep for the prevention of periodontitis.

## Introduction

Periodontitis is a chronic inflammatory condition that begins with the formation of a biofilm by periodontal pathogens and is characterized by the destruction of periodontal tissues (1). In recent years, while tooth loss has decreased as a result of advances in caries prevention and dental care, the number of teeth at risk of developing periodontitis has increased. According to the Survey on the Status of Dental Diseases conducted by the Ministry of Health, Labour and Welfare of Japan, the proportion of people with periodontal pockets increases with age, being present in more than half of adults aged ≥ 45 years.

The onset and progression of periodontitis are associated with oral hygiene behaviors such as brushing habits (2) and dental visits and checkup history (3, 4), as well as conditions such as depression (5, 6), diabetes (7), dyslipidaemia (8, 9), impaired immune function (10, 11), smoking habits (12, 13), obesity (14), drug use (15) and alcohol consumption (16, 17), socioeconomic factors, including educational attainment (18) and income (18, 19), psychosocial stress (20–22), sleep deprivation, dietary habits (23, 24) and postmenopausal hormonal changes (25, 26).

Among these factors, sleep plays a crucial role in regulating systemic inflammatory responses and immune function, and its association with periodontitis has attracted increasing attention. Several cross-sectional studies have reported a higher prevalence of periodontitis among individuals with short sleep duration (27, 28). For example, a population-based cross-sectional study reported that individuals with sleep deficiency of <7 h had higher odds of periodontitis than those with an adequate sleep duration of 7–8 h, even after adjustment for potential confounders (odds ratio [OR] = 1.19, 95% CI: 1.06–1.38, 29). However, the findings of previous studies have not been consistent. A previous meta-analysis found no significant overall association between short sleep duration and periodontitis (OR = 1.04, 95% CI: 0.83–1.29), whereas sleep duration of ≤5 h was significantly associated with periodontitis (OR = 1.41, 95% CI: 1.33–1.51) (27). Differences in sleep-duration thresholds, sleep assessment methods, periodontal outcomes, and study populations may partly account for these inconsistent findings. However, because many of these studies have been cross-sectional, the temporal relationship between short sleep duration and the subsequent development of periodontitis remains difficult to clarify. Furthermore, it has not been sufficiently investigated whether short sleep duration precedes the development or progression of periodontitis, or whether periodontitis and its related factors adversely affect sleep.

Given this background, we hypothesized that short sleep duration is associated with the subsequent development of self-reported severe periodontitis. Using longitudinal data from a nationwide prospective Internet cohort study—the 2023–2025 waves of the Japan COVID-19 and Society Internet Survey (JACSIS)—the present study aimed to examine the longitudinal association between sleep duration and self-reported incident severe periodontitis among individuals without self-reported severe periodontitis at baseline.

## Materials and methods

### Study design and data source

This prospective cohort study used data from the 2023–2025 waves of the Japan COVID-19 and Society Internet Survey (JACSIS), a nationwide internet-based survey described elsewhere (30). The 2023 survey served as the baseline, and follow-up data from 2024 and 2025 were used for longitudinal analyses.

### Participants

In this study, we used respondents to the 2023 JACSIS as our baseline cohort. Invalid responses were excluded according to predefined JACSIS criteria. Furthermore, participants who were determined to have severe periodontitis (score ≥2) at baseline were excluded. Participants who completed the 2024 and 2025 follow-up surveys were included in the final analysis. This observational study used existing JACSIS data, and no prior sample size planning was conducted.

The study protocol was approved by the Osaka International Cancer Institute Research Ethics Committee (date of approval: June 19, 2020; approval No.: 20084) and the Ethics Review Board of Okayama University (date of approval: July 5, 2024; approval No.: 2408–009; date of approval: January 19, 2024; approval No.: 2403–044).

### Survey items

#### Severe periodontitis

The presence of severe periodontitis was assessed using a self-administered questionnaire-based screening method (31). The validity of this screening approach has previously been confirmed in a Japanese population (31). The assessment utilized the following four question items:

1. “Do you think you might have gum disease?” (Yes/No)
2. “Have your teeth ever become loose on their own?” (Yes/No)
3. “Have you ever been told by a dentist or dental hygienist that you are losing bone around your teeth?” (Yes/No)
4. “Have your gums bled in the past 3 months?” (Sometimes, Often, Always/Never, Rarely)

For each item, a score of 1 was assigned for answers of “Yes” or “Sometimes, Often, Always” and a score of 0 for all other responses. Then, a total score was calculated, with ≥2 points defined as severe periodontitis (31). The onset of periodontitis was defined as the first transition from non-severe to severe during the follow-up period.

#### Covariates

Age and sex were included as basic covariates. Age was treated as a continuous variable, and sex was treated as a binary variable (male or female).

#### Socioeconomic factors

##### Occupation

Occupation was classified as: technical/professional, clerical, sales and marketing, service industry, security, manufacturing/machine assembly, transportation/machine operation, construction/mining, transportation/cleaning/packaging, other and unemployed.

##### Educational history

We classified the highest level of education attained into the following categories: junior high school, high school (public or private), vocational school, junior college or technical college, university (public, private, or national) and graduate school. We then converted these classifications into years of education based on the International Standard Classification of Education (32) and the Japanese education system, and treated them as continuous variables.

##### Household annual income

Household annual income was assessed by selecting one of 19 income brackets (in million Japanese yen [JPY]) and classified into the following categories: <3, ≥3 to <6, ≥6 to <9 and ≥9. Responses of “Prefer not to answer” or “Do not know” were treated as missing values.

##### Sleep duration

Average daily sleep duration over the past month was assessed and classified into < 6 h and ≥ 6 h. The response “Do not know” was treated as a missing value. Short sleep was defined as < 6 h (33, 34).

##### Dental visits and checkups

We assessed whether participants had undergone dental visits for treatment purposes (e.g., treatment of caries) within the past year and treated this as a binary variable (“Yes/No”). We also assessed whether participants had undergone dental checkups (e.g., regular dental examinations, professional cleaning or scaling), excluding visits for treatment purposes, within the past year. This was also treated as a binary variable (“Yes/No”). Based on these variables, participants were categorized into four groups: (1) dental checkups only, (2) dental treatment visits only, (3) both dental checkups and treatment visits and (4) neither. Because these items were not included in the 2025 survey, we used baseline (2023) data and treated them as covariates.

##### Comorbidities

Participants were classified into one of three categories regarding diabetes, dyslipidaemia, immune disorders or conditions associated with impaired immune function, and depression: “currently present,” “previously present” or “absent.” Note that because immune disorders or conditions associated with impaired immune function were not included in the 2025 survey, baseline (2023) data were used as covariates.

##### Smoking habit

Participants were classified as non-smokers, former smokers or current smokers.

##### Alcohol consumption

Daily alcohol intake (in sake equivalents [*gō*]) was assessed and classified as “none,” “< 2” or “≥ 2.” Consumption of ≥ 2 *gō* corresponds to ≥ 40 g of pure alcohol per day and is classified as high-risk drinking (17, 35).

##### Drug use

History of cannabis use was classified as “none,” “past” or “current.”

##### Physical measurements

Body mass index (BMI) was calculated from height and weight and used as a continuous variable. To account for the influence of extreme outliers, physiologically implausible BMI values (< 15 and > 50) were excluded (36). Because BMI was not included in the 2025 survey, we treated it as a covariate using baseline (2023) data.

### Statistical analysis

In this study, to examine the association between sleep duration and self-reported incident severe periodontitis, a Cox proportional hazards model was conducted as the primary analysis. Cox regression was selected because the primary outcome was time to first self-reported incident severe periodontitis during follow-up. Participants without self-reported severe periodontitis at baseline (2023) were followed for up to 2 years until the first self-reported incident severe periodontitis or censoring. Because periodontal status was assessed annually, event time was assigned to the first survey wave in which self-reported severe periodontitis was observed. Participants who first met the self-reported severe periodontitis definition in 2024 were treated as having an event at year 1, and those who first met the definition in 2025 were treated as having an event at year 2. Participants who did not develop self-reported severe periodontitis during the follow-up period were censored at their last observation. Participants with missing covariate data were excluded using complete-case analysis.

In the Cox proportional hazards model, hazard ratios (HRs) and 95% confidence intervals (CIs) were calculated, and a robust variance estimator was used to address heteroscedasticity in the model. The assumption of proportional hazards was verified using Schoenfeld residuals. The following covariates were adjusted for: age, sex, years of education, BMI (2023), smoking history, alcohol consumption, diabetes, dyslipidaemia, depression, immune disorders (2023), household income, dental visits and dental checkups (both 2023) and occupation.

As a secondary analysis, GEE with a logit link, binomial distribution and exchangeable correlation structure was used to analyze repeated measures of periodontal status and assess the robustness of the Cox model findings.

To explore the potential influence of over-adjustment on the association between short sleep duration and self-reported incident severe periodontitis, we conducted a stepwise model comparison as a sensitivity analysis. First, we constructed a crude model that included sleep duration only. Next, Model A was adjusted for age, sex, educational attainment, smoking history, alcohol consumption, household income, the combined categories of dental visits and dental checkups, and occupation, but did not include candidate mediators. Finally, Model B was additionally adjusted for diabetes, dyslipidaemia, depression, immune-related conditions, and BMI. We compared changes in the association between short sleep duration and self-reported incident severe periodontitis across these three models. All three models were estimated using the same complete-case sample with robust standard errors. This analysis was intended to explore the potential influence of over-adjustment and did not constitute a rigorous causal mediation analysis.

All statistical analyses were performed using Stata (version 19; StataCorp LLC, College Station, TX, United States). Effect estimates are presented with 95% CIs and corresponding *p*-values.

### Ethics approval

The study protocol was approved by the Osaka International Cancer Institute Research Ethics Committee (date of approval: June 19, 2020; approval No.: 20084) and the Ethics Review Board of Okayama University (date of approval: January 19, 2024; approval No.: 2403–044; date of approval: July 5, 2024; approval No.: 2408–009).

## Results

### Participant selection

A flowchart of participant selection is shown in Figure 1. Ultimately, 8,144 individuals were traceable at all three time points and included in the final analysis.

**Figure 1:**
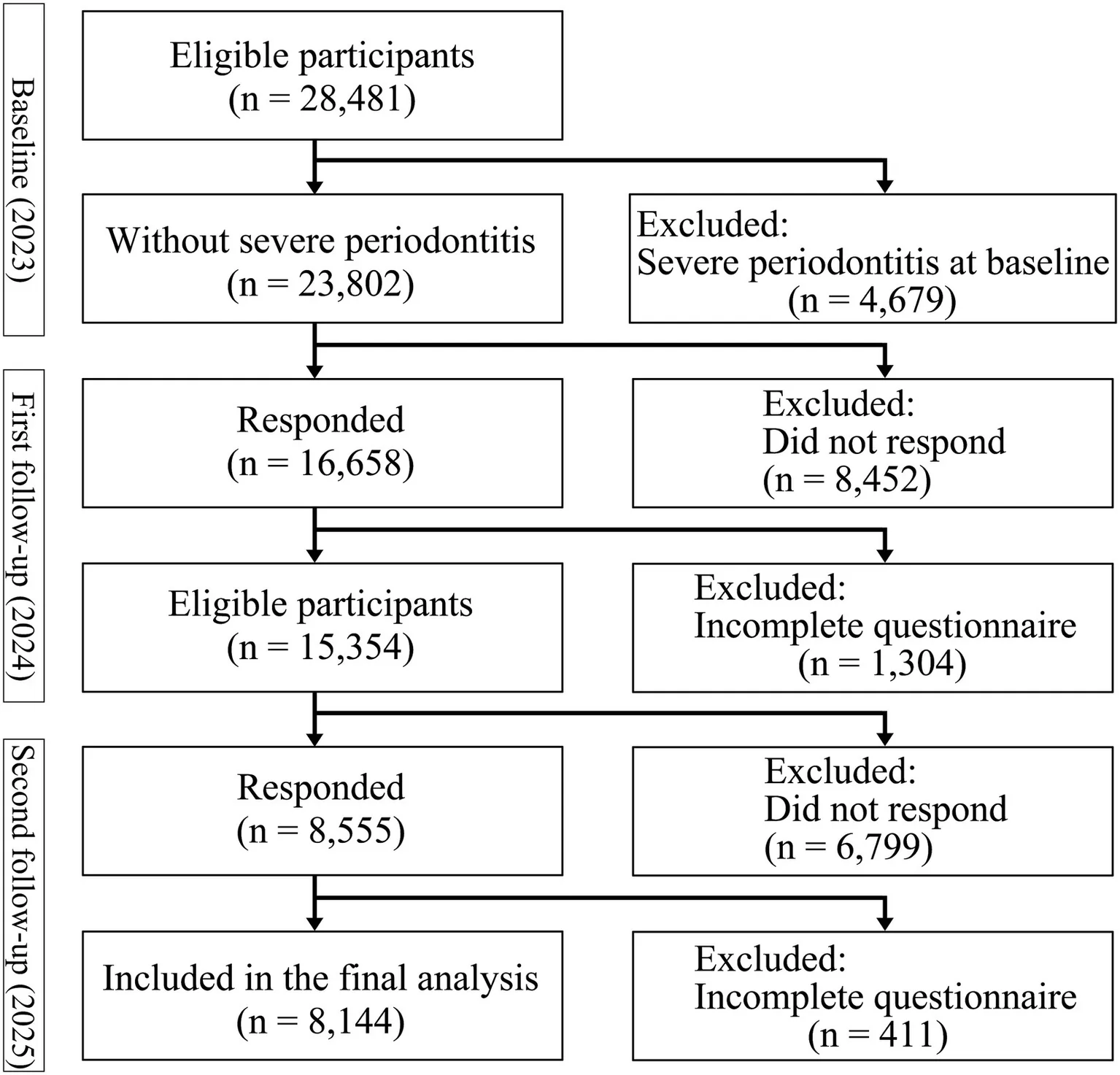
Flowchart of participant selection. A total of 28,481 valid respondents were identified from the 2023 Japan COVID-19 and Society Internet Survey (JACSIS). After excluding participants with self-reported severe periodontitis at baseline (*n* = 4,679), 23,802 individuals were eligible for follow-up. Among these individuals, 16,658 responded to the 2024 survey, 15,354 of whom provided valid responses (1,304 invalid responses were excluded). Of these, 8,555 responded to the 2025 survey, and 8,144 provided valid responses (411 invalid responses were excluded). These 8,144 participants were included in the final analysis.

### Baseline characteristics

Table 1 summarizes the baseline characteristics of the 8,144 participants. The mean age was 49.9 (SD 16.7) years, 47.4% were women, and 22.7% reported sleeping <6 h per night.

**Table 1:** Baseline characteristics of participants without self-reported severe periodontitis in 2023 (*n* = 8,144).

Variable	Total
Age, mean (SD)	49.9 (16.7)
BMI, mean (SD)	22.0 (3.6)^*^
Years of education, mean (SD)	14.7 (2.0)^*^
Sex, n (%)	
Male	4,285 (52.6)
Female	3,859 (47.4)
Sleep duration, *n* (%)	
≥6 h	6,197 (77.3)
<6 h	1,817 (22.7)
Household income, *n* (%)^*^	
≥9 million JPY	1,170 (18.0)
6–9 million JPY	1,460 (22.4)
3–6 million JPY	2,485 (38.2)
<3 million JPY	1,390 (21.4)
Smoking status, *n* (%)	
Never	5,365 (65.9)
Past	1,412 (17.3)
Current	1,367 (16.8)
Alcohol consumption, *n* (%)	
None	1,244 (15.3)
<2 go/day	5,671 (69.6)
≥2 go/day	1,229 (15.1)
Diabetes, *n* (%)	
None	7,552 (92.7)
Past	110 (1.4)
Current	482 (5.9)
Dyslipidaemia, *n* (%)	
None	6,682 (82.1)
Past	375 (4.6)
Current	1,087 (13.4)
Depression, *n* (%)	
None	7,204 (88.5)
Past	529 (6.5)
Current	411 (5.1)
Immune disorder, *n* (%)	
None	7,904 (97.1)
Past	99 (1.2)
Current	141 (1.7)
Dental visits and dental checkups (2023), *n* (%)	
Dental checkups only	533 (6.5)
Treatment visits only	344 (4.2)
Both	4,048 (49.7)
Neither	3,219 (39.5)
Occupation, *n* (%)	
Professional/technical	1,225 (15.0)
Clerical	1,349 (16.6)
Sales	681 (8.4)
Service	445 (5.5)
Security	61 (0.8)
Manufacturing	248 (3.1)
Transport	97 (1.2)
Construction	75 (0.9)
Cleaning/packaging	154 (1.9)
Other	1,029 (12.6)
Unemployed	2,780 (34.1)

Values are presented as mean (SD) or number (%). Percentages were calculated using available data; therefore, denominators may vary due to missing values. All variables were assessed at baseline in 2023. Participants with severe periodontitis at baseline were excluded.

^*^Data may be missing; percentages were calculated using available data, therefore denominators may vary.

Percentages may not sum to 100 due to rounding.

Descriptive statistics for the 23,802 participants without self-reported severe periodontitis as of 2023, along with comparisons with the final analysis cohort (*n* = 8,144) and those excluded from the analysis (*n* = 15,658), are shown in Supplementary Table S1. The final analysis cohort was slightly older than the excluded group, but the distributions of major covariates were generally similar.

### Cox proportional hazards model

We conducted a Cox proportional hazards analysis among 8,144 individuals without severe periodontitis at baseline in 2023, with the first self-reported incident severe periodontitis during the 2023–2025 follow-up period as the outcome (Table 2); 6,688 individuals were included in the analysis. Sleeping <6 h was associated with a higher risk of self-reported incident severe periodontitis compared with sleeping ≥6 h (HR = 1.28, 95% CI: 1.13–1.46, *p* < 0.001). Increasing age, lower income, diabetes, dyslipidaemia, depression and smoking were also associated with a higher risk. No clear association was observed for sex or alcohol consumption. Compared with participants who underwent only a dental examination, those who underwent only dental treatment were associated with a higher risk (HR = 1.60, 95% CI: 1.14–2.24, *p* = 0.007). By contrast, no clear difference was observed between participants who underwent both a dental examination and treatment and those who underwent neither.

**Table 2:** Cox proportional hazards model for incident severe periodontitis (2023–2025).

Variable	HR	95% CI	*p*-value
Sleep duration (ref: ≥6 h)			
<6 h	1.28	1.13–1.46	<0.001
Demographic factors			
Age (per year)	1.01	1.00–1.01	<0.001
Female (vs male)	1.01	0.88–1.16	0.896
Education (years)	1.00	0.97–1.03	0.998
Socioeconomic status			
Income (ref: ≥9 million JPY)			
6–9 million JPY	1.11	0.92–1.35	0.280
3–6 million JPY	1.22	1.02–1.45	0.029
<3 million JPY	1.27	1.04–1.54	0.018
Lifestyle factors			
BMI (2023)	1.01	0.99–1.02	0.426
Smoking status (ref: never)			
Past	1.32	1.13–1.55	<0.001
Current	1.31	1.12–1.53	0.001
Alcohol consumption (ref: none)			
<2 go/day	1.06	0.94–1.21	0.346
≥2 go/day	1.09	0.90–1.30	0.375
Systemic conditions			
Diabetes (ref: none)			
Past	1.82	1.38–2.41	<0.001
Current	1.24	1.02–1.50	0.027
Dyslipidaemia (ref: none)			
Past	1.53	1.25–1.88	<0.001
Current	1.22	1.04–1.42	0.014
Depression (ref: none)			
Past	1.52	1.25–1.85	<0.001
Current	1.87	1.53–2.30	<0.001
Immune disorder (2023) (ref: none)			
Past	0.94	0.58–1.53	0.806
Current	1.22	0.89–1.69	0.220
Dental visits and dental checkups (ref: dental checkups only)			
Treatment visits only	1.60	1.14–2.24	0.007
Both	1.14	0.88–1.49	0.326
Neither	1.04	0.79–1.37	0.757
Occupation (ref: professional/technical)			
Clerical	1.16	0.94–1.43	0.168
Sales	1.22	0.96–1.57	0.109
Service	1.32	0.99–1.76	0.057
Security	0.90	0.45–1.80	0.769
Manufacturing	1.56	1.13–2.15	0.006
Transport	1.54	0.96–2.46	0.074
Construction	1.64	0.94–2.85	0.080
Cleaning/packaging	1.48	1.03–2.12	0.034
Other	1.14	0.89–1.45	0.300
Unemployed	1.04	0.84–1.29	0.705

Hazard ratios (HRs) and 95% confidence intervals (CIs) were estimated using Cox proportional hazards regression models with robust variance estimators clustered at the individual level. The outcome was incident self-reported severe periodontitis during the follow-up period from 2023 to 2025. All models were restricted to participants without self-reported severe periodontitis at baseline in 2023. All covariates were measured at baseline (2023). Proportional hazards assumptions were assessed using Schoenfeld residuals. Number of participants included in the model: 6,688.

### Generalized estimating equations (GEEs)

As a secondary analysis, GEEs were used to model the transition from non-severe to severe self-reported periodontitis as a repeated-measure outcome. Sleeping <6 h per night was associated with a higher likelihood of transition (OR = 1.26, 95% CI: 1.09–1.46, *p* = 0.002). The results for other covariates were broadly consistent with those from the Cox model (Table 3).

**Table 3:** Factors associated with the transition from non-severe to severe self-reported periodontitis status among participants without self-reported severe periodontitis at baseline.

Variable	OR	95% CI	*p*-value
Sleep duration (ref: ≥6 h)			
<6 h	1.26	1.09–1.46	0.002
Demographic factors			
Age (per year)	1.01	1.01–1.02	<0.001
Female (ref: male)	0.97	0.83–1.14	0.712
Education (years)	1.00	0.96–1.03	0.947
Socioeconomic status			
Income (ref: ≥9 million JPY)			
6–9 million JPY	1.13	0.92–1.40	0.250
3–6 million JPY	1.19	0.98–1.45	0.077
<3 million JPY	1.30	1.04–1.62	0.021
Lifestyle factors			
BMI (2023)	1.01	0.99–1.03	0.411
Smoking status (ref: never)			
Past	1.40	1.17–1.67	<0.001
Current	1.43	1.19–1.72	<0.001
Alcohol consumption (ref: none)			
<2 go/day	1.06	0.92–1.22	0.389
≥2 go/day	1.09	0.89–1.34	0.390
Systemic conditions			
Diabetes (ref: none)			
Past	1.59	1.11–2.28	0.012
Current	1.31	1.04–1.64	0.021
Dyslipidaemia (ref: none)			
Past	1.49	1.19–1.86	<0.001
Current	1.22	1.03–1.45	0.025
Depression (ref: none)			
Past	1.77	1.42–2.20	<0.001
Current	2.18	1.69–2.80	<0.001
Immune disorder (2023) (ref: none)			
Past	1.14	0.63–2.05	0.669
Current	1.25	0.83–1.87	0.279
Dental visits and dental checkups (ref: dental checkups only)			
Treatment visits only	1.77	1.19–2.64	0.005
Both	1.14	0.84–1.54	0.392
Neither	1.07	0.78–1.46	0.687
Occupation (ref: professional/technical)			
Clerical	1.16	0.92–1.45	0.211
Sales	1.29	0.98–1.69	0.065
Service	1.31	0.95–1.80	0.097
Security	1.06	0.54–2.06	0.865
Manufacturing	1.62	1.12–2.33	0.010
Transport	1.41	0.82–2.42	0.217
Construction	1.50	0.76–2.97	0.247
Cleaning/packaging	1.31	0.87–1.97	0.199
Other	1.13	0.88–1.45	0.341
Unemployed	0.99	0.79–1.26	0.958

Odds ratios (ORs) and 95% confidence intervals (CIs) were estimated using generalized estimating equations (GEE) with a binomial distribution and logit link. All models were restricted to participants without self-reported severe periodontitis at baseline in 2023. GEE models accounted for repeated observations within individuals using an exchangeable working correlation structure and robust standard errors. Number of participants included in the model: 6,713.

### Exploratory analysis of potential mediators

To explore potential influence of over-adjustment on the association between short sleep duration and self-reported incident severe periodontitis, we compared a crude model with models excluding and including the potential mediators (Supplementary Table S2). In the crude model, short sleep duration was associated with a higher risk of self-reported incident severe periodontitis (HR = 1.38, 95% CI: 1.21–1.57). The association remained statistically significant in Model A, which excluded the potential mediators (HR = 1.36, 95% CI: 1.20–1.55). After additional adjustment for diabetes, dyslipidaemia, depression, immune-related conditions and BMI in Model B, the estimate was slightly attenuated but remained statistically significant (HR = 1.28, 95% CI: 1.13–1.46).

### Additional descriptive analysis

We have provided a supplementary description of the changes in self-reported periodontal status regarding severe periodontitis among the 8,144 participants who were trackable at all three time points (Supplementary Table S3).

## Discussion

In the present study, we used data from JACSIS, a nationwide prospective cohort study, to examine the association between sleep duration and the risk of self-reported incident severe periodontitis. Short sleep duration (<6 h per night) was associated with a higher risk of self-reported incident severe periodontitis compared with longer sleep duration, even after adjustment for socioeconomic factors, lifestyle habits and systemic conditions.

These findings are consistent with previous research demonstrating an association between short sleep duration and periodontitis. Previous cross-sectional studies have reported a higher prevalence of periodontitis among individuals with short sleep duration (27, 28). Zhou et al. (27) reported no significant overall association between short sleep duration and periodontitis (OR = 1.04, 95% CI: 0.83–1.29), or between short sleep duration and severe periodontitis (OR = 0.94, 95% CI: 0.75–1.16). However, sleep duration of ≤5 h was significantly associated with periodontitis (OR = 1.41, 95% CI: 1.33–1.51). In addition, a population-based cross-sectional study reported that individuals with sleep deficiency (<7 h) had higher odds of periodontitis than those with adequate sleep (7–8 h) after adjustment for potential confounders (OR = 1.19, 95% CI: 1.06–1.38) (29). In the present study, short sleep duration (<6 h) was associated with a higher risk of self-reported incident severe periodontitis (HR = 1.28, 95% CI: 1.12–1.48). Although direct comparison of these effect estimates is limited by differences in study design, study populations, sleep-duration thresholds, periodontal outcomes, and measures of association, the direction and magnitude of the association observed in the present study were broadly consistent with those reported in some previous studies. However, it has been difficult to clarify the temporal relationship between these factors because previous evidence has been derived primarily from cross-sectional studies. By prospectively following individuals without self-reported severe periodontitis at baseline in 2023, the present study examined the temporal association between short sleep duration and subsequent self-reported incident severe periodontitis. Thus, the present findings add to the limited longitudinal evidence and complement the evidence derived primarily from cross-sectional studies.

Although biological mechanisms were not directly assessed, altered circadian regulation, inflammatory responses, oxidative stress and metabolic changes may explain the observed association. Short sleep duration may disrupt circadian regulation and neuroendocrine function, which may in turn contribute to systemic inflammation and immune dysregulation. These changes may alter the host response to periodontal pathogens and increase susceptibility to periodontal tissue inflammation and destruction (37). Sleep is closely linked to melatonin secretion through the regulation of the circadian rhythm, and sleep deprivation is known to lead to a decrease in melatonin secretion (38, 39). Melatonin possesses antioxidant and immunomodulatory properties and has been reported to contribute to the suppression of inflammatory cytokines and the reduction of oxidative stress (40). Periodontitis is a chronic inflammatory condition, and because excessive immune responses and oxidative stress are involved in the destruction of periodontal tissues, it is conceivable that reduced melatonin levels due to sleep deprivation may contribute to the exacerbation of inflammation in periodontal tissues (41). In addition, circadian disruption may be linked to periodontitis through oxidative stress and systemic inflammation (42). Short sleep duration has been associated with impaired glucose metabolism, insulin resistance, and obesity (43). These metabolic alterations, together with dyslipidaemia, may in turn contribute to periodontal inflammation (7–9, 14). These mechanisms are likely to be interrelated and may collectively explain the observed association. On the other hand, the relationship between sleep and periodontal health may also be bidirectional. Although short sleep duration may contribute to periodontal inflammation through the pathways described above, periodontitis and its associated systemic inflammation may, in turn, adversely affect sleep duration or quality (44). In the future, further studies are required to explore the bidirectional relationship.

Furthermore, sleep deprivation is associated with reduced insulin sensitivity (45–48) and increased psychological stress (22, 49, 50), and it has been suggested that these factors may influence the development of periodontitis. In the present study, because an association was observed even after adjusting for these factors as covariates, the association between short sleep duration and self-reported incident severe periodontitis cannot be fully explained by these pathways alone, suggesting that it may arise through complex biological pathways.

To explore the potential pathways underlying this association further, we conducted additional analyses comparing models that included potential mediating factors—such as diabetes, dyslipidaemia, depression, immune-related diseases and BMI—with models that did not include them. The association between short sleep duration and self-reported incident severe periodontitis was slightly attenuated after adjusting for these variables, but remained statistically significant. These findings suggest that additional adjustment for these potential mediators did not materially alter the observed association, although these factors may partially explain it. It should be noted that the included variables may act as both confounders and mediators. Therefore, these results should be interpreted as exploratory, and further research using more detailed measurements is needed to elucidate the underlying mechanisms.

In this study, in addition to short sleep duration, diabetes, dyslipidaemia, depression, and past and current smoking were associated with self-reported incident severe periodontitis. Previous studies have reported associations between depression and periodontal disease (5, 6), diabetes and periodontal disease (7), dyslipidaemia and periodontal disease (8, 9), and smoking and periodontal disease (12, 13). Individuals who reported dental visits but no dental checkups had a higher risk of self-reported incident severe periodontal disease than those who reported dental checkups but no dental visits. This finding may reflect the possibility that dental visits were prompted by existing oral symptoms or treatment needs, whereas dental checkups were preventive. Participants who reported neither dental checkups tended to be younger than the other groups (Supplementary Table S4), which may partly explain the lack of an increased risk in this group. However, these were secondary findings, and information on the reasons for dental attendance was unavailable and periodontal status was not clinically assessed; therefore, these findings should be interpreted cautiously.

From a public health perspective, sleep duration may be a modifiable risk factor for periodontal disease. Insufficient sleep is widespread, particularly among the working-age population (51), and is a factor that can be addressed through behavioral interventions and improvements to the workplace environment. Therefore, promoting improvements in sleep habits could serve as a new approach to complement existing periodontal disease prevention measures. Because sleep deprivation and periodontal disease share common underlying factors, such as socioeconomic factors and lifestyle habits, public health policies that promote healthy sleep may also contribute to improving the oral health of the general population.

This study has several strengths. First, we used data from a large-scale, longitudinal nationwide study. Second, we adopted a prospective cohort design, which allowed us to examine the temporal relationship with self-reported incident severe periodontitis.

However, several limitations should be noted. First, both sleep duration and severe periodontitis were assessed by self-report. Although the questionnaire has been validated, self-reported periodontal status is subject to measurement error, and some misclassification of incident severe periodontitis is therefore possible. By defining the event as the first transition from non-severe to severe self-reported status, we used a consistent event definition. However, because some participants later returned to non-severe status, outcome misclassification due to self-reported assessment remains possible. Second, fluctuations in periodontal status were observed between survey waves, which may include the influence of subjective perception; however, we partially mitigated this effect by focusing on the initial self-reported incident severe periodontitis. Third, the possibility of unmeasured confounding cannot be ruled out, particularly as detailed oral hygiene habits were not sufficiently adjusted for. Fourth, because this study was based on an Internet survey, the possibility of selection bias cannot be ruled out. However, JACSIS was conducted on a nationwide scale, and the number of participants was maintained across each survey wave through replacement sampling (30). Furthermore, as shown in Supplementary Table S1, the distribution of short sleep duration was similar between included and excluded participants, whereas included participants were older and more likely to be male. Therefore, selection bias due to loss to follow-up cannot be ruled out. Fifth, because the study population was limited to participants in an online survey, the generalizability of the results is somewhat limited. However, because JACSIS was conducted on a national scale and employed sampling that accounted for population distribution, it is considered to reflect the general adult population of Japan to a certain extent.

## Conclusion

The results of the present nationwide prospective cohort study indicated that shorter sleep duration was associated with an increased risk of developing self-reported severe periodontitis. While these results suggest that sleep duration may be an important lifestyle factor in periodontal health, further research using clinical periodontal assessments and intervention studies is needed to clarify the causal relationship and underlying mechanisms.

## Data Availability

The data analyzed in this study is subject to the following licenses/restrictions: the data used in the present study are not deposited in a public repository because they contain personally identifiable or potentially sensitive information. In accordance with ethical guidelines in Japan, the dissemination of these data is restricted by the Research Ethics Committee of the Osaka International Cancer Institute. Any inquiries regarding data use should be directed to Takahiro Tabuchi (tabuchitak@gmail.com). More details regarding data availability can be found on the JACSIS website (https://jacsis-study.jp/dug/index.html; accessed on December 11, 2025). Requests to access these datasets should be directed to Takahiro Tabuchi, tabuchitak@gmail.com.
