# Supplementary material for: Short sleep duration and incident self-reported severe periodontitis among Japanese adults: a nationwide prospective cohort study
Source: Front Public Health. 2026 Sep 30;14:1946230. doi: 10.3389/fpubh.2026.1946230 (PMC13661196; doi:10.3389/fpubh.2026.1946230)
Supplement: Supplementary file 1 [file Table_1.DOCX]

Supplementary Table S1. Baseline characteristics by inclusion in the final analytical sample (attrition analysis)

| Variable | All eligible (n = 23,802) | Included (n = 8,144) | Excluded (n = 15,658) |
| --- | --- | --- | --- |
| **Age, mean (SD)** | 47.10 (18.03) | 49.87 (16.73) | 45.67 (18.51) |
| **BMI, mean (SD)^*^** | 21.93 (3.52) | 22.00 (3.57) | 21.90 (3.50) |
| **Years of education, mean (SD)^*^** | 14.53 (1.97) | 14.68 (1.97) | 14.45 (1.97) |
| **Sex, n (%)** |  |  |  |
| Male | 11,514 (48.4) | 4,285 (52.6) | 7,229 (46.2) |
| Female | 12,288 (51.6) | 3,859 (47.4) | 8,429 (53.8) |
| **Sleep duration, n (%)** |  |  |  |
| ≥6 hours | 17,760 (76.2) | 6,197 (77.3) | 11,563 (75.6) |
| <6 hours | 5,557 (23.8) | 1,817 (22.7) | 3,740 (24.4) |
| **Household income, n (%)^*^** |  |  |  |
| ≥9 million JPY | 3,271 (18.1) | 1,170 (18.0) | 2,101 (18.1) |
| 6–9 million JPY | 4,037 (22.3) | 1,460 (22.4) | 2,577 (22.2) |
| 3–6 million JPY | 6,913 (38.2) | 2,485 (38.2) | 4,428 (38.1) |
| <3 million JPY | 3,897 (21.5) | 1,390 (21.4) | 2,507 (21.6) |
| **Smoking status, n (%)** |  |  |  |
| Never | 16,373 (68.8) | 5,365 (65.9) | 11,008 (70.3) |
| Former | 3,787 (15.9) | 1,412 (17.3) | 2,375 (15.2) |
| Current | 3,642 (15.3) | 1,367 (16.8) | 2,275 (14.5) |
| **Alcohol consumption, n (%)** |  |  |  |
| None | 4,282 (18.0) | 1,244 (15.3) | 3,038 (19.4) |
| <2 go/day | 16,104 (67.7) | 5,671 (69.6) | 10,433 (66.6) |
| ≥2 go/day | 3,416 (14.4) | 1,229 (15.1) | 2,187 (14.0) |
| **Diabetes, n (%)** |  |  |  |
| None | 22,136 (93.0) | 7,552 (92.7) | 14,584 (93.1) |
| Past | 357 (1.5) | 110 (1.4) | 247 (1.6) |
| Current | 1,309 (5.5) | 482 (5.9) | 827 (5.3) |
| **Dyslipidemia, n (%)** |  |  |  |
| None | 19,786 (83.1) | 6,682 (82.0) | 13,104 (83.7) |
| Past | 1,036 (4.4) | 375 (4.6) | 661 (4.2) |
| Current | 2,980 (12.5) | 1,087 (13.3) | 1,893 (12.1) |
| **Depression, n (%)** |  |  |  |
| None | 20,972 (88.1) | 7,204 (88.5) | 13,768 (87.9) |
| Past | 1,651 (6.9) | 529 (6.5) | 1,122 (7.2) |
| Current | 1,179 (5.0) | 411 (5.0) | 768 (4.9) |
| **Immune disorder, n (%)** |  |  |  |
| None | 23,007 (96.7) | 7,904 (97.1) | 15,103 (96.5) |
| Past | 369 (1.6) | 99 (1.2) | 270 (1.7) |
| Current | 426 (1.8) | 141 (1.7) | 285 (1.8) |
| **Dental visit (2023), n (%)** |  |  |  |
| No | 10,945 (46.0) | 3,752 (46.1) | 7,193 (45.9) |
| Yes | 12,857 (54.0) | 4,392 (53.9) | 8,465 (54.1) |
| **Dental checkup (2023), n (%)** |  |  |  |
| No | 10,537 (44.3) | 3,563 (43.8) | 6,974 (44.5) |
| Yes | 13,265 (55.7) | 4,581 (56.3) | 8,684 (55.5) |
| **Occupation, n (%)** |  |  |  |
| Professional / technical | 3,676 (15.4) | 1,225 (15.0) | 2,451 (15.7) |
| Clerical | 3,990 (16.8) | 1,349 (16.6) | 2,641 (16.9) |
| Sales | 2,052 (8.6) | 681 (8.4) | 1,371 (8.8) |
| Service | 1,433 (6.0) | 445 (5.5) | 988 (6.3) |
| Security | 212 (0.9) | 61 (0.7) | 151 (1.0) |
| Manufacturing | 822 (3.5) | 248 (3.0) | 574 (3.7) |
| Transport | 284 (1.2) | 97 (1.2) | 187 (1.2) |
| Construction | 198 (0.8) | 75 (0.9) | 123 (0.8) |
| Cleaning/packaging | 473 (2.0) | 154 (1.9) | 319 (2.0) |
| Other | 3,035 (12.8) | 1,029 (12.6) | 2,006 (12.8) |
| Unemployed | 7,627 (32.0) | 2,780 (34.1) | 4,847 (31.0) |

* Percentages were calculated using available data; therefore denominators may vary due to missing values.

Supplementary Table S2. Exploratory analysis of potential mediators in the association between short sleep duration and self-reported incident severe periodontitis

| **Model** | **HR (95% CI)** | **p-value** |
| --- | --- | --- |
| Crude model | 1.38 (1.21-1.57) | <0.001 |
| Model A (without potential mediators) | 1.36 (1.20-1.55) | <0.001 |
| Model B (with potential mediators) | 1.28 (1.13-1.46) | <0.001 |

The crude model included sleep duration only. Model A was adjusted for age, sex, educational attainment, smoking history, alcohol consumption, household income, the combined categories of dental visits and dental checkups, and occupation. Model B was additionally adjusted for diabetes, dyslipidaemia, depression, immune-related conditions, and BMI. All models were estimated using the same complete-case sample. This exploratory analysis was conducted to examine the potential influence of over-adjustment and does not constitute a formal causal mediation analysis. CI, confidence interval; HR, hazard ratio.

Supplementary Table S3. Transitions in self‑reported severe periodontitis status between survey waves (2023–2025)

| **Transition pattern (2023 → 2024 → 2025)** | **n** | **%** |
| --- | --- | --- |
| No → No → No | 6,881 | 84.5 |
| No → No → Yes | 403 | 4.9 |
| No → Yes → No | 531 | 6.5 |
| No → Yes → Yes | 329 | 4.0 |
| **Total** | **8,144** | **100.0** |

No = self‑reported non‑severe periodontitis

Yes = self‑reported severe periodontitis

Severe periodontitis status was assessed using a self‑reported questionnaire with previously validated items. Transitions reflect changes in self‑reported status between survey waves and do not necessarily indicate clinical progression or regression of periodontitis. Therefore, in the main analysis, progression was defined as the first transition from non‑severe to severe status.

Supplementary Table S4. Baseline characteristics according to dental visit and checkup categories

| Variable | Checkup only | Visit only | Both | Neither | p-value |
| --- | --- | --- | --- | --- | --- |
| Age, mean (SD) | 48.1 (16.6) | 51.8 (16.0) | 54.4 (16.7) | 48.5 (16.3) | <0.001 |
| BMI, mean (SD) | 21.8 (3.4) | 22.2 (3.8) | 22.0 (3.5) | 22.0 (3.7) | 0.075 |
| Years of education, mean (SD) | 15.0 (1.9) | 14.8 (1.9) | 14.8 (1.9) | 14.6 (2.0) | <0.001 |
| Sex, n (%) |  |  |  |  |  |
| Male | 522 (49.0) | 386 (56.1) | 3,992 (49.3) | 3,670 (57.0) | <0.001 |
| Female | 544 (51.0) | 302 (43.9) | 4,104 (50.7) | 2,768 (43.0) |  |
| Sleep duration, n (%) |  |  |  |  |  |
| ≥6 hours | 841 (79.5) | 521 (76.4) | 6,395 (79.7) | 4,843 (76.6) | <0.001 |
| <6 hours | 217 (20.5) | 161 (23.6) | 1,631 (20.3) | 1,481 (23.4) |  |
| Household income, n (%) |  |  |  |  |  |
| ≥9 million JPY | 203 (23.6) | 102 (18.1) | 1,417 (21.5) | 889 (17.5) | <0.001 |
| 6–9 million JPY | 202 (23.5) | 114 (20.2) | 1,498 (22.8) | 1,133 (22.3) |  |
| 3–6 million JPY | 288 (33.5) | 227 (40.3) | 2,359 (35.9) | 1,862 (36.6) |  |
| <3 million JPY | 167 (19.4) | 120 (21.3) | 1,302 (19.8) | 1,197 (23.6) |  |
| Smoking status, n (%) |  |  |  |  |  |
| Never | 766 (71.9) | 429 (62.4) | 5,343 (66.0) | 4,194 (65.1) | <0.001 |
| Past | 186 (17.4) | 130 (18.9) | 1,517 (18.7) | 1,006 (15.6) |  |
| Current | 114 (10.7) | 129 (18.8) | 1,236 (15.3) | 1,238 (19.2) |  |
| Alcohol consumption, n (%) |  |  |  |  |  |
| None | 490 (46.0) | 300 (43.6) | 3,782 (46.7) | 3,164 (49.1) | <0.001 |
| <2 go/day | 457 (42.9) | 327 (47.5) | 3,383 (41.8) | 2,386 (37.1) |  |
| ≥2 go/day | 119 (11.2) | 61 (8.9) | 931 (11.5) | 888 (13.8) |  |
| Diabetes, n (%) |  |  |  |  |  |
| None | 995 (93.3) | 615 (89.4) | 7,356 (90.9) | 5,929 (92.1) | 0.002 |
| Past | 12 (1.1) | 11 (1.6) | 145 (1.8) | 119 (1.8) |  |
| Current | 59 (5.5) | 62 (9.0) | 595 (7.3) | 390 (6.1) |  |
| Dyslipidemia, n (%) |  |  |  |  |  |
| None | 864 (81.1) | 518 (75.3) | 6,253 (77.2) | 5,432 (84.4) | <0.001 |
| Past | 42 (3.9) | 42 (6.1) | 459 (5.7) | 308 (4.8) |  |
| Current | 160 (15.0) | 128 (18.6) | 1,384 (17.1) | 698 (10.8) |  |
| Depression, n (%) |  |  |  |  |  |
| None | 955 (89.6) | 601 (87.4) | 7,256 (89.6) | 5,777 (89.7) | 0.006 |
| Past | 73 (6.8) | 49 (7.1) | 515 (6.4) | 348 (5.4) |  |
| Current | 38 (3.6) | 38 (5.5) | 325 (4.0) | 313 (4.9) |  |
| Immune disorder, n (%) |  |  |  |  |  |
| None | 1,042 (97.7) | 664 (96.5) | 7,826 (96.7) | 6,276 (97.5) | <0.001 |
| Past | 8 (0.8) | 14 (2.0) | 92 (1.1) | 84 (1.3) |  |
| Current | 16 (1.5) | 10 (1.5) | 178 (2.2) | 78 (1.2) |  |
| Occupation, n (%) |  |  |  |  |  |
| Professional/technical | 227 (21.3) | 107 (15.6) | 1,378 (17.0) | 1,027 (16.0) | <0.001 |
| Clerical | 223 (20.9) | 139 (20.2) | 1,537 (19.0) | 1,188 (18.5) |  |
| Sales | 62 (5.8) | 57 (8.3) | 611 (7.5) | 620 (9.6) |  |
| Service | 55 (5.2) | 49 (7.1) | 402 (5.0) | 377 (5.9) |  |
| Security | 3 (0.3) | 4 (0.6) | 78 (1.0) | 49 (0.8) |  |
| Manufacturing | 35 (3.3) | 19 (2.8) | 255 (3.1) | 277 (4.3) |  |
| Transport | 6 (0.6) | 9 (1.3) | 59 (0.7) | 92 (1.4) |  |
| Construction | 9 (0.8) | 8 (1.2) | 28 (0.3) | 69 (1.1) |  |
| Cleaning/packaging | 17 (1.6) | 18 (2.6) | 143 (1.8) | 161 (2.5) |  |
| Other | 117 (11.0) | 72 (10.5) | 866 (10.7) | 757 (11.8) |  |
| Unemployed | 312 (29.3) | 206 (29.9) | 2,739 (33.8) | 1,821 (28.3) |  |

Data are presented as mean (standard deviation) for continuous variables and number (percentage) for categorical variables. Differences across groups were assessed using analysis of variance (ANOVA) for continuous variables and the chi-square test for categorical variables. All tests were two-sided. All variables were assessed at baseline in 2023. Percentages were calculated using available data; therefore, denominators may vary due to missing values.
